# Experimental investigation into the functionality of alkaline water electrolysis with ion-solvating membrane in anode feed mode using diluted potassium hydroxide

**DOI:** 10.1016/j.heliyon.2025.e42075

**Published:** 2025-01-17

**Authors:** Dieter Jarosch, John James Warren, Jörg Kapischke

**Affiliations:** Ansbach University of Applied Sciences, Ansbach, 91522, Germany

**Keywords:** Anion exchange membrane water electrolysis, Ion-solvating membrane, Permselectivity, Diluted potassium hydroxide solution as electrolyte, Electrolyte concentration changes, Electrolyte flow rate

## Abstract

This study explores the unique operating behavior of an alkaline water electrolysis cell equipped with an ion-solvating membrane, operated with a diluted alkaline electrolyte, specifically 1-M potassium hydroxide (1M KOH), in anode feed mode. Our investigations reveal several key insights.

**Charge transport:**

In an ion-solvating membrane, charge transport occurs through both the cations and anions of the electrolyte. Due to electro-osmosis, cation transport to the cathode results in a combined hydrogen-electrolyte discharge from the cathode compartment of the electrolysis cell. The discharged electrolyte is more concentrated than the electrolyte supplied to the anode. The concentration and flow rate of the electrolyte increase with current density and electrolyte temperature.

**Current density dependence:**

Since only a fraction of the total charge is transferred by hydroxide ions within the membrane, current density strongly depends on the electrolyte flow through the anode compartment.

**Membrane stability and performance:**

The membrane's mechanical and chemical stability enables operation at high temperatures, up to 80 °C. This stability enables increased current density at a given cell voltage.

**Effects of catalyst use:**

Using cathode catalysts with high surface areas, such as Raney-Ni, enhances current density because highly concentrated liquid potassium hydroxide forms at the cathode during operation. Anode catalysts with high surface areas increase current density, but only if the flow of hydroxide ions is not impeded. Otherwise, the *jV*-curve exhibits transport-limited behavior.

## Highlights

• Alkaline electrolysis is affected by cation transport in the anion exchange membrane.
• An alkaline water electrolysis cell with an ion-solvating membrane can also be efficiently operated with a diluted potassium hydroxide solution in anode feed mode.
• Ion-solvating membranes produce a strong alkali in addition to hydrogen, which enables the use of Raney-Ni as a hydrogen evolution catalyst.
• Temporal electrolyte changes affect current density.

## 1 Introduction

In the future, green hydrogen, produced by electrolysis of water using renewable energy, will increasingly be used as a climate-neutral fuel. This requires an increase in the resource-efficient production of electrolysers by using abundant and inexpensive raw materials. Alkaline electrolysis appears particularly economical, as many electrode metals exhibit corrosion resistance due to their passivity in an alkaline environment. Consequently, a wide choice of suitable catalysts for the evolution of hydrogen and oxygen exist, eliminating the need for rare earth elements or expensive precious metals. Currently, iron and nickel are the primary metals used for this purpose [1].

A durable separator is required for safe gas separation of hydrogen and oxygen. Ideally, this separator should not impede the water electrolysis process and should have very low electrical resistance. To meet these criteria, the separator must be as thin as possible while maintaining high ionic conductivity. With “Nafion”, a mechanically and chemically stable separator with high proton conductivity has already been developed for acidic environments. Now, similar polymer membranes are being researched for alkaline conditions. To date however, sufficiently stable anion exchange membranes (AEM) with high anion selectivity and conductivity have yet to be realized.

The AEMs developed so far lack the required stability in concentrated potassium hydroxide, limiting their use to environments with mildly alkaline conditions with a pH range of 9–11 and temperatures below 60 °C [2,3]. Additionally, current membranes swell to different extents in pure water and potassium hydroxide solutions of varying concentrations, compromising their operational lifespan, particularly in anode feed mode. This makes achieving a stable membrane coating with a catalyst and ionomer, necessary for high current densities, extremely challenging. For example, the well-known AEM membrane from Fumatech, FAA3-50, shows a length increase of approximately 27 % between water and 1-M KOH.

The stability issues observed in AEMs are not present in homogeneous, pore-free ion-solvating membranes (ISM). These membranes ensure reliable gas separation between hydrogen and oxygen [[4], [5], [6]]. Fumatech's FAAM-10 is an example of such a membrane. It can be manufactured very thin (10 μm) and is stable in hot (80 °C) concentrated potassium hydroxide. Normally, this membrane requires a concentrated (approx. 6-molar) potassium hydroxide solution to maintain high ionic conductivity and minimize ohmic resistance [7]. The specific conductivity of a 6-M potassium hydroxide solution is approximately three times higher than that of a 1-M solution at the same temperature [8]. However, handling hot concentrated potassium hydroxide solutions is extremely hazardous, complicating and increasing the cost of electrolysis operations due to its aggressive and corrosive nature.

To address this issue, this work demonstrates that electrolysis with such a membrane can also be conducted using a diluted 1-M potassium hydroxide solution in anode feed mode. During electrolysis, the concentration of the liquid alkaline electrolyte in the cathode chamber increases. This allows the use of Raney-Ni as a hydrogen evolution catalyst, which significantly increases the electrochemically active surface area, thereby improving the efficiency of the electrolyzer. On the anode side, any deficiency of hydroxide ions can be compensated for by increasing the pump feed of the dilute alkaline electrolyte. The use of a catalyst for oxygen evolution to further increase efficiency must not hinder the transport of the necessary hydroxide ions to the reaction site. Raney-Ni is unsuitable as an anode catalyst because the high diffusion resistance hinders the transport of hydroxide ions to the reaction site. This results in a significantly transport-limited *jV*-curve.

The use of diluted potassium hydroxide as an electrolyte enables operation in a less hazardous and aggressive environment. The components on the anode side only need to be corrosion-resistant to diluted potassium hydroxide. In contrast, the demister on the cathode side must be suitable for concentrated potassium hydroxide, but it can be smaller due to the lower amount of condensed potassium hydroxide. These factors help reduce the overall system cost.

## 2 Experimental setup and materials

### 2.1 Structure of the electrolysis cell

**Fig. 1:**
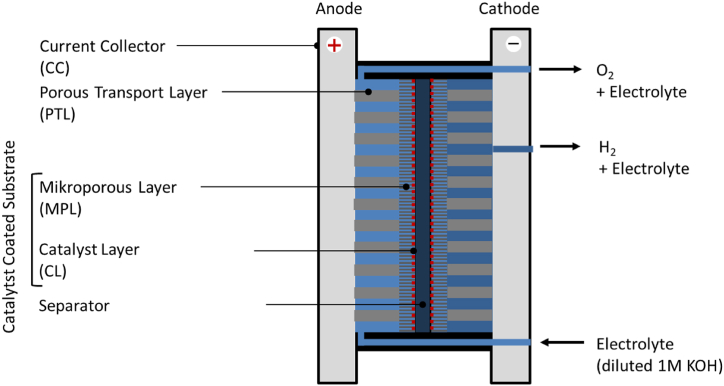
General structure of the electrolysis cell, operated in anode feed mode.

All experiments were performed using a commercial Acta electrolysis cell enclosure. This cell is designed to operate exclusively with an alkaline electrolyte flowing through the anode compartment. Fig. 1 shows the general structure of the cell.

### 2.2 Materials of the electrolysis cell

All materials of the cell should withstand high pH conditions. Although a dilute 1-M solution of potassium hydroxide (1M KOH) serves as the electrolyte on the anode side, concentration changes can occur during operation.

#### 2.2.1 Current collector

A stainless steel plate serves as the current collector for the anode side, while nickel-plated steel is used for the cathode side.

#### 2.2.2 Porous transport layer (PTL)

**Fig. 2:**
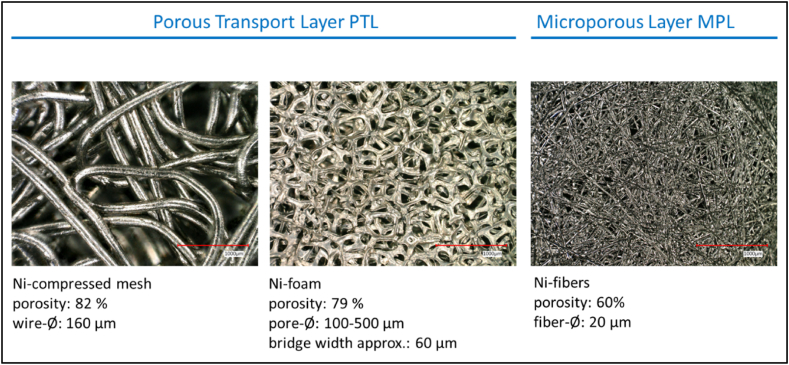
Microscopic images of porous transport layers and microporous layer [9].

On the cathode side, a 3 mm thick Ni-foam with a porosity of 79 % serves as the porous transport layer (PTL). In contrast, the PTL on the anode side must allow for the least obstructed electrolyte flow possible (2–50 l/h). Therefore, a 3 mm thick compressed Ni mesh with a wire diameter of 160 μm and a porosity of 82 % is used (Fig. 2). The images illustrate the different material structures, giving an impression of the flow resistance to the electrolyte.

#### 2.2.3 Microporous layer (MPL)

A 100 μm thin layer of sintered Ni fibers (Ø: 20 μm) from the company Bekaert functions as a protective layer for the membrane and as a catalyst substrate for anode and cathode.

#### 2.2.4 Catalyst layer

It should be noted that no emphasis was placed on optimizing the catalysts for the hydrogen and oxygen evolution reactions, particularly in terms of achieving a highly active catalytic surface. In fact, the Ni fibers of the MPL act as their own catalyst for both the hydrogen and oxygen evolution reactions. However, they are sensitive to changes in electrical potentials, especially during start-up and shutdown, and to the presence of hydrogen, oxygen, or contaminants in the electrolyte, particularly iron. Therefore, the MPL was additionally coated with a thin catalytic layer primarily to improve the reproducibility of the measurements. To this end, the catalyst synthesis was deliberately kept as simple as possible.

##### 2.2.4.1 Preparation of the anode catalyst layer

A simple method is described in Ref. [10]. After cleaning the 100 μ MPL sheet (Ø: 100 mm) in 15 wt% HCl solution for 30 min, it was then exposed to 40 ml of a 20 mM Fe(NO_3_)_3_ · 9H_2_O solution for 1 min at room temperature. Following this procedure, amorphous Ni-Fe hydroxide should be deposited on the MPL.

##### 2.2.4.2 Preparation of the cathode catalyst layer

A NiMo alloy highly catalyzes the hydrogen evolution reaction (HER). An electrochemical deposition method is described in Ref. [11]. After cleaning the 100 μm MPL sheet (Ø: 90 mm) in a 15 wt% HCl solution for 30 min, a 2-min cathodic deposition of NiMo alloy at −80 mA/cm^2^ was performed using a 200 ml bath solution with graphite as the counter electrode. The bath solution contained 7.9 g of NiSO_4_ · 6H_2_O, 8.8 g of Na_3_C_6_H_5_O_7_· 2H_2_O, and 4.8 g of Na_2_MoO_4_, adjusted to pH 10.5 with ammonia solution. All chemical reagents were of analytical grade.

##### 2.2.4.3 Raney-Ni

To test the influence of highly catalytic active surfaces on the efficiency of the electrolysis cell with the FAAM-10 membrane, a commercial Raney-Ni NIH33/PTFE mixture from the company gaskatel was used as cathode catalyst [12]. The original MPL made from Ni fibers provided insufficient adhesion for the catalyst. Therefore, Ni foam 5763, with a porosity of 96 %, from Recemat was rolled to a thickness of 100 μm and used as a catalyst support. A mixture of 55 mg/cm^2^ was sprinkled onto the cathode MPL and 44 mg/cm^2^ onto the anode MPL, then solidified by hand using a stainless steel roller.

##### 2.2.4.4 Conditioning procedure for Raney-Ni NiH33

**Fig. 3:**
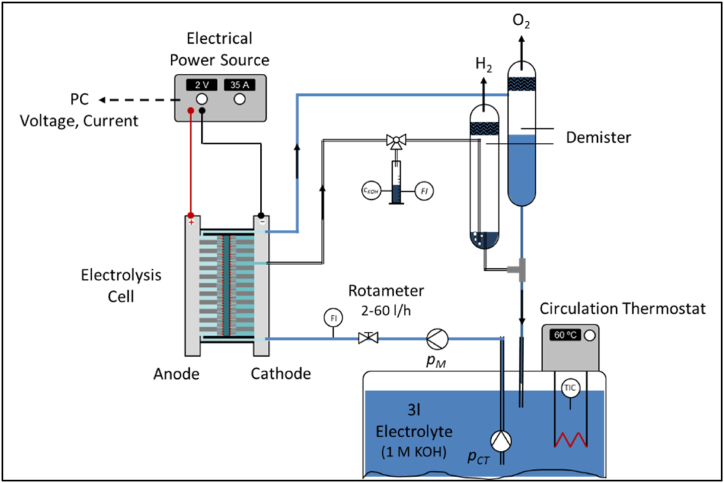
Test bench for recording jV-curves of the electrolysis cell.

Raney-Ni is pyrophoric, meaning it has a very large specific surface area and would spontaneously combust in air, forming electrically non-conductive oxides. To handle it in air, it was passivated and coated with a very thin oxide layer. This layer must be reduced, for example, over 4 h at a constant cathodic current density of 50 mA/cm^2^ at an elevated temperature of 60 °C. After the reduction process, the cathodic Raney-Ni layer must not be exposed to air. To prevent this, the hydrogen outlet of the electrolysis cell is blocked using the electrolyte (Fig. 3).

#### 2.2.5 Separator

An anion-exchange membrane, FAAM-10 with a thickness of 10 μm, from the german company Fumatech was used.

##### 2.2.5.1 Activation procedure

The membrane (Ø: 100 mm) was treated with a 6 M of potassium hydroxide solution (technical grade) for 3 days [13] in a petri dish at room temperature and ambient air. After this treatment, the membrane exhibited minimal swelling in diameter and was installed directly into the cell enclosure in its wet state.

### 2.3 Test bench

The test bench for recording current density-voltage curves (*jV-*curves) is shown in Fig. 3. The device used as a constant voltage source is the EA Automatik PSI 8080 40 T. The electrolyte is 1 M potassium hydroxide (KOH, technical grade), which circulates through the anode compartment at a flow rate of 2–60 L/h. During operation, oxygen is produced at the anode and must be separated from the electrolyte using a demister. Additionally, small amounts of concentrated electrolyte are discharged along with hydrogen at the cathode, necessitating the use of a second separator. The hydrogen outlet is immersed in the liquid electrolyte to prevent air entry, which is essential if Raney-Ni is used as the cathode catalyst. A three-way valve allows switching between a measuring separator and a measuring cylinder to register the flow and take samples of the discharged electrolyte. The concentration of the discharged electrolyte at the cathode was determined by means of density measurement, based on the relationships given in Handbook of Chemistry and Physics [14].

The gas purity of the electrolytically produced hydrogen was not the focus of this work. However, due to the extremely thin FAAM-10 membrane, the critical oxygen content in the hydrogen stream was monitored intermittently using a simple detonation test. In all test series, this test was negative, indicating that the oxygen content in the hydrogen was less than 5 vol-% at atmospheric pressure [15].

## 3 Test results of the electrolysis cell with the FAAM-10 membrane

Except for the experiments using Raney-Ni as the electrode catalyst, all test series were conducted with a NiFe coating as the anode catalyst and a NiMo coating as the cathode catalyst.

### 3.1 Stationary jV-curve

**Fig. 4:**
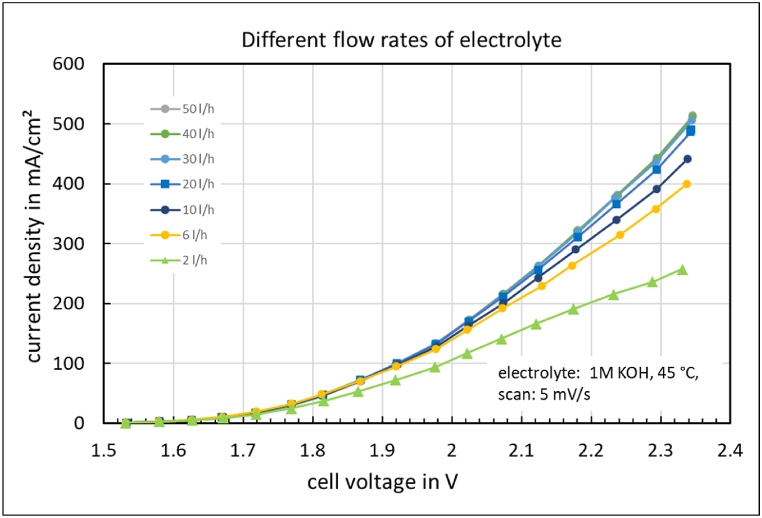
Quasi-stationary jV-curve of the cell with FAAM-10 membrane at different flow rates of the electrolyte.

The quasi-stationary *jV*-curve of the electrolysis cell in unpressurized operation at different flow rates of the electrolyte is shown in Fig. 4. The cell produces wet hydrogen, meaning that a liquid is discharged from the cathode compartment along with hydrogen throughout the electrolysis process. The quasi-stationary *jV*-curves were recorded 10 min after the start-up of the electrolyzer at a constant temperature.

### 3.2 Current density as a function of the electrolyte flow rate

**Fig. 5:**
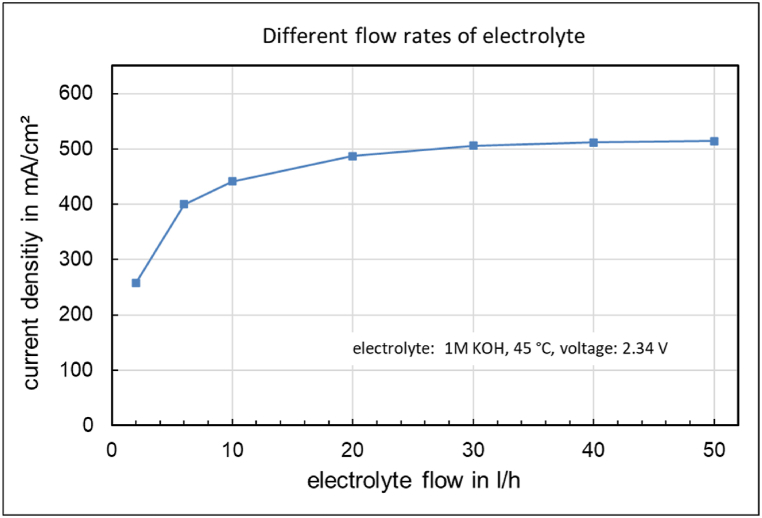
Current density as a function of the electrolyte flow rate at constant voltage.

The flow of the dilute 1 M KOH electrolyte is adjusted at a constant cell voltage. The results are shown in Fig. 5 and indicate that the current density is strongly dependent on the electrolyte flow.

### 3.3 Start-up and long-term behavior under constant cell voltage

**Fig. 6:**
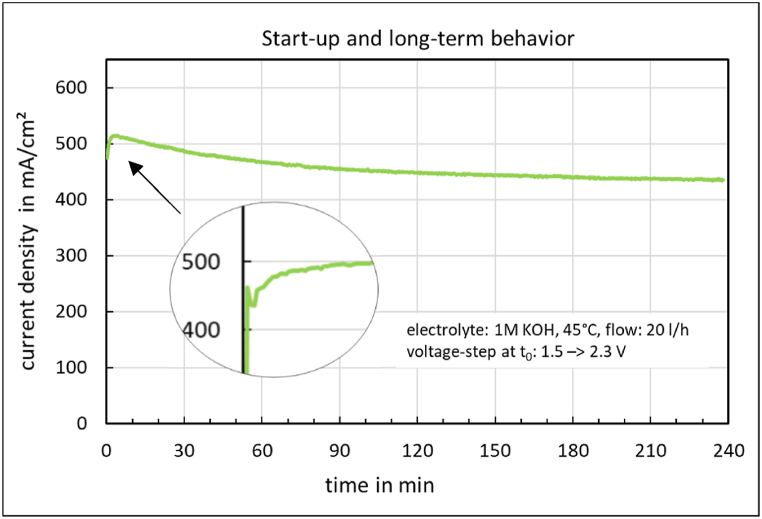
Start-up and long-term behavior of the electrolysis cell with FAAM-10 membrane.

The start-up and long-term behavior of the electrolysis cell is shown in Fig. 6. A non-Faraday current density peak, caused by the double layer capacity of the electrodes [16], can be observed after the sudden voltage change from 1.5 V to 2.34 V. The current density then increases and reaches its maximum value after 2 min. Subsequently, the current density decreases by approximately 100 mA/cm^2^ over 4 h.

**Fig. 7:**
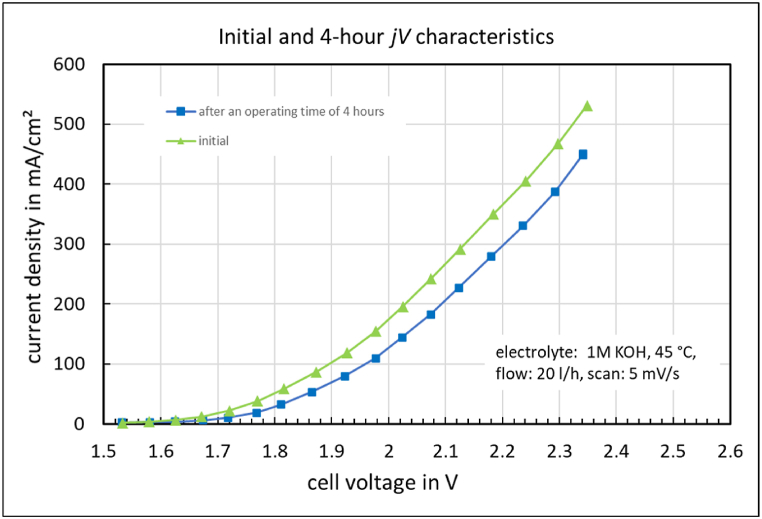
jV characteristics at the initial time and after 4 h of electrolysis.

The *jV* characteristics at the initial time and after 4 h of electrolysis are shown in Fig. 7.

### 3.4 Discharge and change in concentration of the potassium hydroxide solution in the cathode compartment

**Fig. 8:**
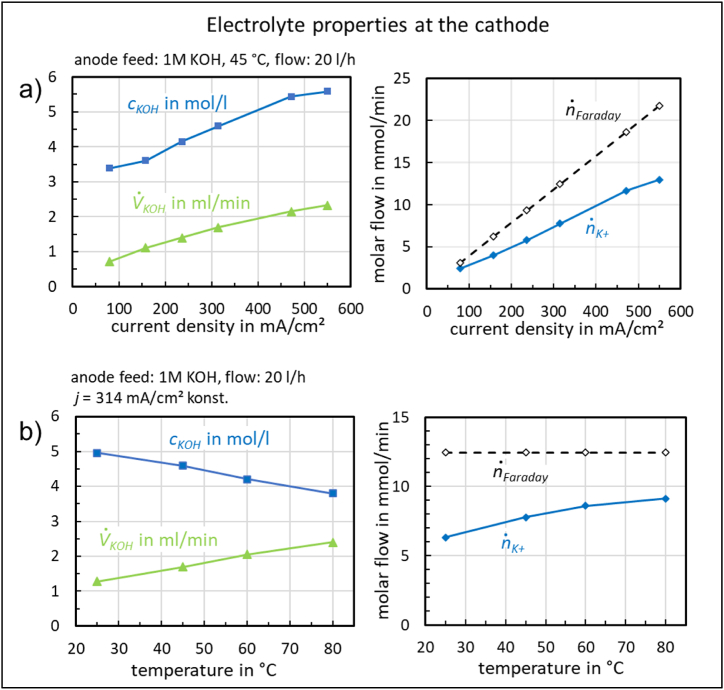
Concentration, volume flow and molar fluxes of the electrolyte discharged from the cathode compartment as a function of current density (a) and temperature (b).

The concentrations *c*_*KOH*_ of the potassium hydroxide, volume flows $\dot{V}$_*KOH*_ and molar fluxes $\dot{n}$_*K+*_*,* $\dot{n}$_*Faraday*_ discharged as a function of current density are shown in Fig. 8 (a).

The positive potassium ion current ($\dot{n}$_*K+*_*)* discharged at the cathode compartment carries part of the total charge transferred ($\dot{n}$_*Faraday*_) during the electrolysis reaction, with the results shown in Table 1.

**Table 1:** Percentage contribution of potassium ion current to the total charge transferred as a function of current density at 45 °C.

current density in mA/cm^2^	79	157	236	314	472	550
$\dot{n}$_*K+*__/_$\dot{n}$_*Faraday*_ in %	79	65	62	63	63	60

The values can be calculated using Faraday's law:

Faraday hydroxide ion current at each current level: $\dot{n}$_*OH-*_ = $\dot{n}$_*Faraday*_ = *I/(z F)*

*z*: valence = 1, *F*: Faraday constant = 96485 As/mol.

Potassium ion current discharged from the cathode compartment: $\dot{n}$_*K*+_ = *c*_*KOH*_ $\dot{V}$_*KOH*_

Percentage charge of potassium ions: $\dot{n}$_*K+*_*/*$\dot{n}$_*Faraday*_

Tests have shown that the flow of electrolyte from the cathode compartment depends not only on the current density but also on the temperature, as shown in Fig. 8 (b).

The potassium ion current discharged increases with temperature at a constant current density and therefore contributes an increasing proportion of the total charge transferred Table 2.

**Table 2:** Percentage contribution of the potassium ion current to the total charge transferred as a function of temperature at constant current density of 314 mA/cm^2^.

temperature in °C	25	45	60	80
$\dot{n}$_*K+*__/_$\dot{n}$_*Faraday*_ in %	51	63	69	73

### 3.5 Increasing the current density

#### 3.5.1 Influence of the temperature

**Fig. 9:**
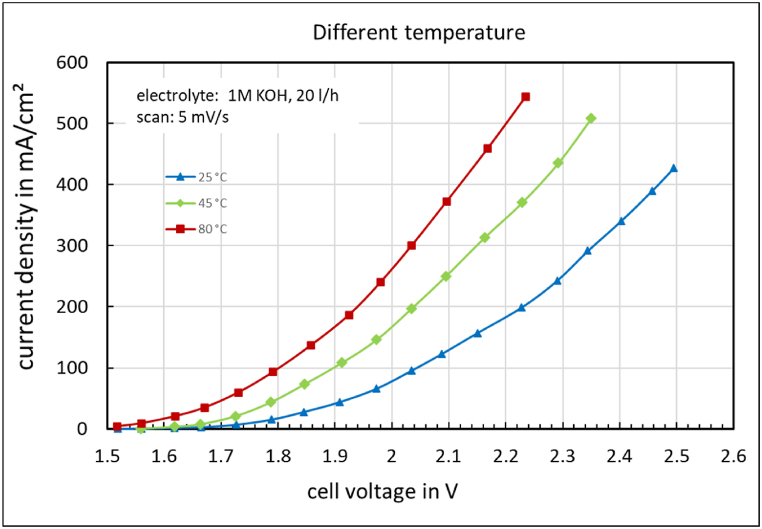
jV-curve of the electrolysis cell at different temperatures.

Fig. 9 shows the influence of temperature on the *jV*-curve of the electrolysis cell. Increasing the temperature leads to an increase in current density at a given cell voltage.

#### 3.5.2 Influence of the catalysts

**Fig. 10:**
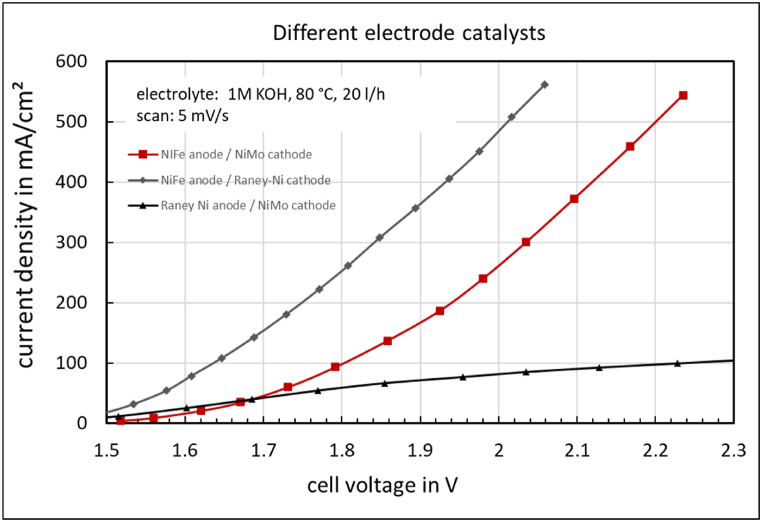
Influence of electrode catalysts on the jV-curve of the electrolysis cell with FAAM-10 membrane.

The influence of different cathode and anode catalysts on the *jV*-curve of the electrolysis cell with FAAM-10 membrane, particularly Raney-Ni, is shown in Fig. 10. When used as a cathode catalyst, Raney-Ni results in a significant increase in current density compared to the NiMo catalyst. However, as an anode catalyst, it causes the current density to decrease dramatically.

## 4 Discussion

### 4.1 Particularities

#### 4.1.1 Anode feed mode

Water is consumed at the cathode of the water electrolysis cell as it reacts to form hydrogen and hydroxide ions.

**Figure:**
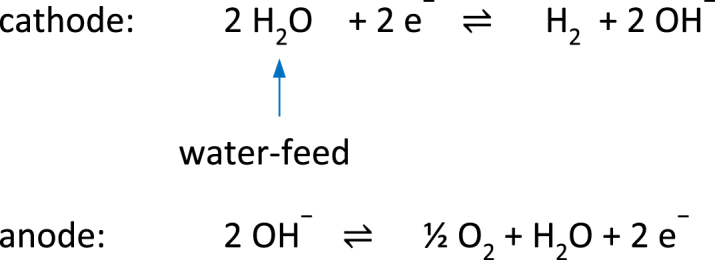

The water feed stream is typically supplied at the point of use to prevent depletion of reactants and enable higher current densities [17,18]. Paradoxically, the electrolysis cell with the Fumatech FAAM-10 membrane achieves higher current densities when water is fed to the anode side.

Water must diffuse across the membrane to the cathode, where it reacts to form hydrogen. In electrolysis cells with anion exchange membranes that have a high selectivity for hydroxide anions e.g., Tokuyama A201, Fumatech FAA-3, dry hydrogen is produced as the product stream [19]. In contrast, the electrolysis cell with the Fumatech FAAM-10 membrane produces wet hydrogen, necessitating the use of a second demister (Fig. 8).

#### 4.1.2 Electrolyte flow rate

Often, the flow rate of the electrolyte is not given much consideration. It must be sufficient to transport water and remove the products and heat. However, in the electrolysis cell with the Fumatech FAAM-10 membrane, a very high flow rate is crucial to achieve a sufficient current density (Fig. 4). A plausible explanation for the strong dependence of the current density on the electrolyte flow rate is the impeded removal of the formed oxygen bubbles. It is therefore expected that the gas bubble transport resistance should also be detectable with a concentrated electrolyte and a comparable oxygen production rate. However, this is not the case. When the electrolysis cell is operated with 6M potassium hydroxide as the anode feed at room temperature, no dependence of the current density on the electrolyte flow rate is observed between 1 and 50 l/h.

#### 4.1.3 Voltage step change

At high overvoltages and current densities, the steady-state *jV* characteristic of an electrolysis cell is usually transport or diffusion limited. A sudden change in voltage results in a large increase in current density, caused by the double layer capacity of the electrodes. The current density then falls back to its steady-state value [16]. In contrast to this behavior, following a non-Faraday current density peak, the current density increases and reaches its maximum value after 2 min (Fig. 6).

#### 4.1.4 Raney-Ni as hydrogen- and oxygen evolution reaction catalyst

Raney nickel is a well-established catalyst for both the hydrogen evolution reaction (HER) and the oxygen evolution reaction (OER). After leaching aluminum from a NiAl alloy, the particles have a high surface area that is accessible to a liquid electrolyte, facilitating the electrochemical reactions. In the electrolysis process examined here using the FAAM membrane, the Raney-Ni only supports hydrogen evolution. Oxygen evolution is significantly inhibited.

#### 4.1.5 Aging

In a new electrolysis cell, it is generally expected that its efficiency will deteriorate over time, meaning the electrical current density at a given cell voltage will decrease. However, a newly constructed electrolysis cell with an FAAM membrane delivers increasingly higher current densities at given voltages with longer operating times, temperature fluctuations, and start-stop operations.

The apparent contradictions can be resolved and explained by considering the function of the anion exchange membrane.

### 4.2 Selectivity of anion exchange membranes

#### 4.2.1 High anion selective exchange membranes (AEM)

**Fig. 11:**
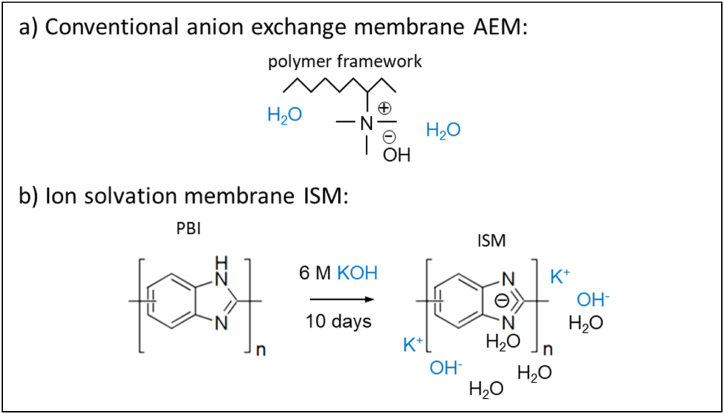
General chemical structure of an AEM with a positively charged ammonium group (a) and ISM (b): Doping of polybenzimidazole with strong alkalis to produce an ISM [13,21].

Conventional anion exchange membranes AEM, e.g. Tokuyama A201, Fumatech FAA-3, have immobile covalently bound cationic functional groups, e.g. positively charged quaternary ammonium groups as shown in Fig. 11 (a) [20].

The ionic conductivity of the membrane is entirely due to mobile anions. Such membranes have high anion permselectivity and are conventionally referred to as AEM.

**Fig. 12:**
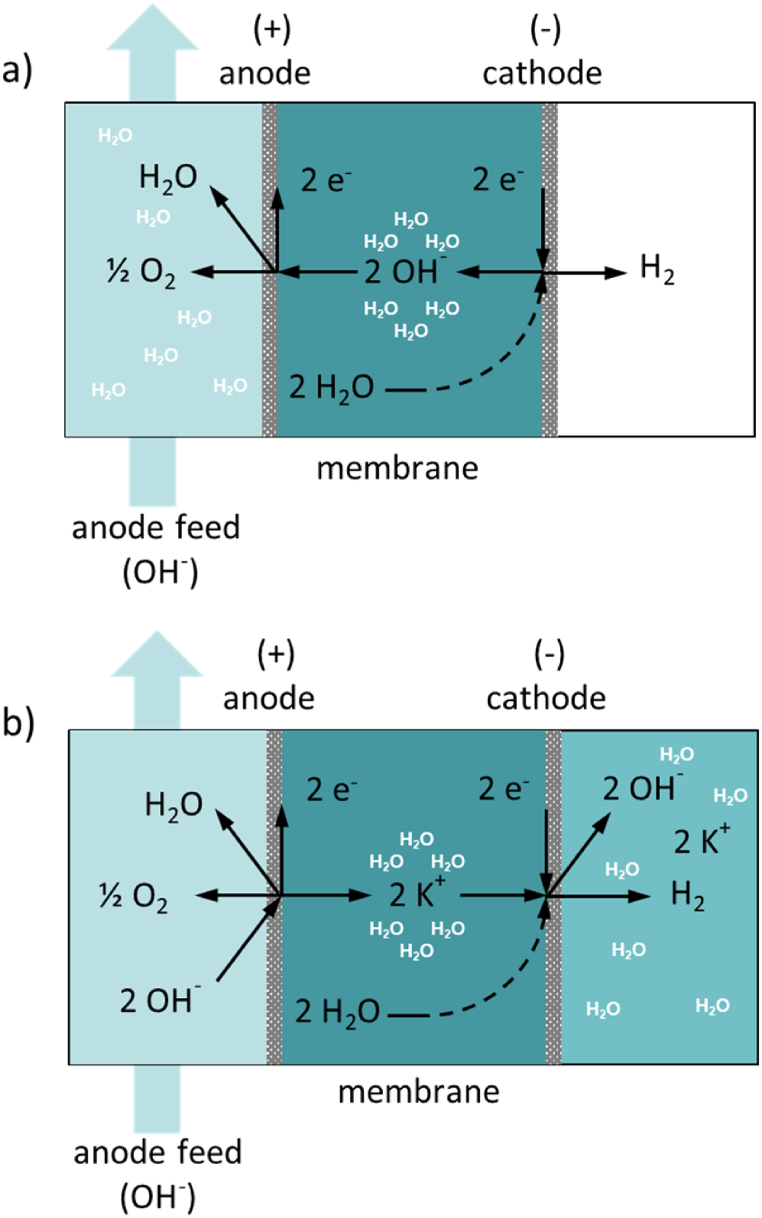
Half-cell reaction at cathode and anode and charge equalization through hydrated OH^−^ anions in alkaline water electrolysis with a membrane of high selectivity for OH^−^ ions (a) and with no selectivity for OH^−^ anions but high selectivity for K^+^ cations (b).

The hydroxide ions required for the oxygen evolution reaction come primarily from the AEM rather than from the dilute potassium hydroxide electrolyte solution fed to the anode compartment. Consequently, the molar transport of the hydroxide ions across the membrane is independent of the electrolyte flow rate through the anode compartment of the electrolysis cell. In anode feed mode, water diffuses through the AEM to the cathode, where it reacts to form hydrogen and hydroxide ions. The hydroxide ions along with their hydration shell, migrate to the anode and react to form oxygen and water. As a result, dry hydrogen is produced at the cathode compartment (Fig. 12 (a)).

Such membranes do not require a liquid electrolyte with high ionic conductivity because the mobile anions in the membrane facilitate charge exchange. Pure water can be supplied to the electrolysis cell. Large pumping capacities are not necessary, and an additional demister for hydrogen-electrolyte separation is not needed [2].

However, these advantages are offset by significant disadvantages. Such membranes have not yet demonstrated long-term corrosion stability in alkaline environments. High concentrations of hydroxide ions damage the membranes [3,22]. They have low mechanical strength and tend to swell significantly, depending on the water and ion concentration of the environment. For example, Fumatech's AEM membrane FAA3-50 shows a length change of 2 % in water and 27 % in 1 M KOH. Concentration differences of the potassium hydroxide across the membrane in anode feed mode lead to significant length changes of the membrane, resulting in high stress on the membrane, which negatively affects the durability of the electrolyzer cell. In contrast, Fumatech's ion-solvating membrane FAAM-10 shows no significant length change in potassium hydroxide of varying concentrations.

#### 4.2.2 Ion solvation membranes (ISM)

Fumatech's FAAM-10 anion exchange membrane is based on polybenzimidazole (PBI) [23,24]. The exact structure and networking have not been disclosed so far. It is obtained by doping polybenzimidazole with strong alkalis, such as 6M KOH, for several days. The anion permselectivity is low. Mobile cations (K^+^) and anions (OH^−^) are responsible for the ionic conductivity of the membrane, as shown in Fig. 11 (b). For this reason, this membrane is also called an ion solvation membrane (ISM).

In contrast to a conventional AEM with high selectivity for OH^−^ anions, the hydroxide anions required for the oxygen evolution reaction come partly from the cathode, as shown in Fig. 12 (a) and partly from the dilute potassium hydroxide electrolyte solution fed to the anode compartment, as shown in Fig. 12 (b) [13].

The half-cell reactions of water electrolysis in an alkaline medium are as follows:

anode (+): 2 OH^−^ ⇌ ½ O_2_ + H_2_O + e^−^

cathode (−): 2H_2_O + 2 e^−^ ⇌ H_2_ + 2 OH^−^

### 4.3 Explanation of the system's unique characteristics

#### 4.3.1 Electrolyte flow rate

The oxygen evolution reaction requires hydroxide ions that must be transported in sufficient quantity to the electrochemically active anode/membrane interface for specific current densities. An adequate supply of hydroxide ions can be provided through the ion-exchange membrane, the supplied anode feed stream, or a combination of both. In the case considered here, only a portion of the hydroxide ions is supplied through the ion-solvating membrane, while the other portion is transported by the feed stream of diluted potassium hydroxide.

A stronger electrolyte flow combined with a porous transport layer with low flow resistance e.g. compressed mesh (Fig. 2) leads to a higher hydroxide ion concentration at the anode/membrane phase boundary, which is necessary for the oxygen evolution, and thus to a higher current density during the electrolysis with an ion-solvating membrane (Fig. 5).

#### 4.3.2 Raney-Ni as hydrogen- and oxygen evolution reaction catalyst

Raney-Ni NiH33 strongly promotes the hydrogen evolution reaction across the entire current density range because of the high hydroxide ion concentration at the cathode and the presence of numerous three-phase boundaries. It also supports the oxygen evolution reaction but only at lower current densities. The resistance for anode feed hydroxide ions to diffuse to the anode/membrane phase boundary is too high, causing the *jV*-curve to be strongly transport-limited (Fig. 10).

#### 4.3.3 Voltage step change

The potassium ions required for charge equalization migrate to the cathode, increasing the concentration of KOH there. This change in solution concentration in the cathode compartment and the associated change in ionic conductivity are responsible for the slow increase in current density after a sudden voltage step change.

Initially, there is 1 M potassium hydroxide solution in the anode and cathode compartments because the electrolyte concentrations equalize by diffusion currents when the cell is not operating. During electrolysis, the concentration of potassium hydroxide in the cathode compartment increases in the first 2 min. This also increases the conductivity of the electrolyte in the cathode compartment and thus the current density at a constant cell voltage.

#### 4.3.4 Aging

After that, the *jV*-curve declines by approximately 100 mA/cm^2^ over 4 h (Fig. 6). This effect has nothing to do with the ion solvation membrane this time. The *jV* characteristics (Fig. 7) before and after long-term operation exhibit the same slope in the ohmic region. Therefore, a different exchange current density must account for this. After shutting down the electrolyzer for over 16 h, the cell voltage decreased from 1.5 V to 0.0 V. Following this procedure, the initial *jV*- curve was restored. This restoration can also be achieved by a 10-min polarity reversal of the cell at −0.5 V. One probable cause could be various activation-deactivation processes affecting the NiFe anode [25]. NiFe facilitates the oxygen evolution reaction (OER). However, during electrolysis operation, Fe goes into solution, causing the catalyst to lose electrochemical activity. During shutdown periods, or accelerated at cell voltages around −0.5 V, the iron redeposits on the anode, regenerating the OER catalyst. This could also explain the increasingly better cell performance with longer operating times and the associated start-stop cycles in the first weeks of operation. The newly produced anode catalyst, and possibly the cathode catalyst as well, is therefore not stable under operating conditions. It changes and even improves over time.

## 5 Conclusions

In this study, the operation of an alkaline electrolysis cell with an ion-solvating membrane was examined. Deviating from the original operation, which fed concentrated potassium hydroxide on both sides of the membrane, a dilute electrolyte flowing only through the anode electrode was used to avoid electrolysis operation in a highly aggressive and corrosive alkaline environment and to reduce costs.

The focus was on investigating the influence of the dilute electrolyte on current density. A strong dependence of the current density on the electrolyte flow rate was observed (Fig. 5). Concentration changes during operation were also measured (Fig. 8), providing insights into the functionality of membrane electrolysis.

### 5.1 Key findings

**Membrane robustness and efficiency:** The ion-solvation membrane, such as the Fumatech FAAM-10, is both chemically and mechanically robust. Very thin membranes can be used, and they are capable of operating in highly alkaline solutions at elevated temperatures up to 80 °C (Fig. 9). Under these conditions, high current densities can be achieved with lower overpotentials.

**Operation with diluted electrolyte:** It is possible to operate the system with diluted potassium hydroxide flowing through the anode electrode, as the potassium hydroxide in the cathode compartment becomes concentrated during electrolysis. This allows the use of a gas diffusion electrode like Raney-Ni NiH33 on the cathode side, enabling a further increase in current density (Fig. 10).

**Catalyst and membrane interface:** The oxygen side requires a stable catalyst with a high electrocatalytic active surface that does not impede the transport of hydroxide ions to the anode/membrane phase boundary.

### 5.2 Future work

The development of a suitable, simple, and cost-effective anode catalyst layer will be the focus of future work. Since the ion-solvating membrane exhibits minimal swelling, a membrane-coating process with a stable anode catalyst, rather than a substrate-coating method, could be promising.

## CRediT authorship contribution statement

**Dieter Jarosch:** Writing – original draft. **John James Warren:** Writing – review & editing. **Jörg Kapischke:** Project administration.

## Data availability statement

Data will be made available on request.

## Ethics approval and consent to participate

Not applicable.

## Declaration of generative AI and AI-assisted technologies in the writing process

During the preparation of this work the authors used ChatGPT (OpenAI) in order to improve readability and language. After using this tool/service, the authors reviewed and edited the content as needed and take full responsibility for the content of the publication.

## Declaration of competing interest

The authors declare that they have no known competing financial interests or personal relationships that could have appeared to influence the work reported in this paper.
